# Magnetic field modulated dynamics of partial discharges in defects of high voltage insulating materials

**DOI:** 10.1038/s41598-022-26675-0

**Published:** 2022-12-21

**Authors:** Marek Florkowski

**Affiliations:** AGH University of Science and Technology, Al. Mickiewicza 30, 30-059 Kraków, Poland

**Keywords:** Electrical and electronic engineering, Energy grids and networks, Power distribution, Power stations

## Abstract

This paper presents original measurement methodology and detection approach to determine the influence of the magnetic field on the partial discharge (PD) dynamics. The application areas refer to insulation systems of both grid and industrial network devices and emerging segments such as high-speed rail, electric vehicles or more electric aircrafts. Conventionally PD measurements are executed only in electric field, however the interaction of magnetic and electric fields influences the dynamics of PDs. The measurement technique allowed to detect quantitatively the effect of magnetic fields on PDs in two representative arrangements: in gaseous void in dielectric material and in corona point-plane setup. Measurements in both configurations have revealed amplification of PDs intensity. The quantitative comparison of PDs evolution in the magnetic field is a novel aspect shown in this paper. Combination of phase resolved images and time-sequence intensity diagrams obtained at magnetic field switching allowed to visualize and determine quantitatively this impact. This effect is attributed to the extension of the charged particle trajectory and the amplification of the electron energy due to acceleration. Thus, the investigated impact of a magnetic field can be perceived as an additional element that influences the PD dynamics.

## Introduction

The electrical insulation of grid and industrial networks devices as well as emerging segments such as high-speed rail, electric vehicles or more electric aircraft is exposed to higher and higher stresses due to increased voltage levels in those applications. The focus of this paper is on original measurement methodology and detection approach to determine the influence of the magnetic field on the partial discharge (PD) dynamics. This is a new research topic, since conventionally PD measurements are executed only in electric field, however the interaction of magnetic and electric fields may influence their behaviour. The high-voltage electric power equipment is constantly exposed to both electric and magnetic fields. It is important to notice that the insulation systems of various grid, substation, rail and industrial network devices such as power transformers, cables, gas-insulated systems and lines, converters, motors, and generators are also exposed to the magnetic fields that are caused by the current flow in conductors; this refers to both AC and DC cases. Since the reliability of electrical power equipment at high- and medium-voltage levels is critical for energy transfers and conversions, advanced design techniques and diagnostic methodologies are being developed. One of the key indicators of high-voltage (HV) insulation quality today is based on the measurement of partial discharges. There are various forms of discharges that are related to the defects that can be found inside or on the surfaces of electrical insulation. In this context, one can typically distinguish internal discharges in tiny air inclusions called voids along with surface discharges or corona discharges. This partial discharge evolution is related to the streamer stages such as inception, channel formation, and development. Streamers are usually interpreted as microchannels of ionized gas and propagate along electric field lines. In the presence of a magnetic field that is superimposed on an electric one, the streamer trajectory is altered due to the additional Lorentz force acting on the charged particles; this leads to a complex circular motion. The propagation of streamers in a very high magnetic field (10 T) was shown in^1^. The experimental observations in this work were focused on tracing a streamer’s trajectory in the presence of a magnetic field. It was shown that the drift motion in crossed electric and magnetic fields is influenced by the Hall angle. Without scattering, electrons move in cycloidal orbits in a direction perpendicular to the electric and magnetic fields. At each scattering event, the electron momentum changes, and a new cycloidal path is taken. The path connecting successive scattering events is forming a trajectory. Imaging observations have shown that the discharge is clearly deflected sideways with an increasing angle at an increasing magnetic field. At higher pressures the streamers branch more often and the velocity of the streamer propagation decreases with pressure. The effects of an alternating magnetic field on a point-plane corona that is measured in an ultra-high frequency (UHF) band were reported in^2^. It was shown that the power spectral density in the corona is reduced in the UHF band of 650–800 MHz in the presence of a magnetic field (250–300 Gauss), for gaps ranging from 15 to 40 mm. The study of a magnetic field on DC corona discharges in a low vacuum was shown in^3^. It was demonstrated that the effect of a magnetic field on a discharge current was most significant with negative corona discharges rather than with positive ones. A magnetically enhanced negative corona that results from field penetration on an ionization region was described in^4^. It was noticed that the relative increase of the discharge current was much larger when the permanent magnets were located near the discharge electrode than near the collecting electrode. The corona inception and breakdown voltage were greatly influenced by the presence of a magnetic field as observed in^5^. It was found that the corona onset voltage and the breakdown voltage decreased with increasing the crossed magnetic field. The influence of a DC magnetic field on PD phase-resolved patterns in a corona cone-plane configuration was discussed in^6^. It has been shown that the magnitude and phase distributions of partial discharge patterns are affected by a perpendicular magnetic field. The influence of an external magnetic field (128 mT) on the statistical parameters of partial discharges in voids was analyzed in^7^. It was found that, although visible differences appear in the PD pattern with and without an applied magnetic field; these cannot be attributed solely to the effects of the Lorenz force. Because PD patterns are state-of-the-art in the diagnostics of high voltage insulation of power equipment today^8–17^, the influence of other factors such as background magnetic fields, similarly to voltage harmonics^18^ are essential for the proper interpretation of any measurement results. The influence of the longitudinal magnetic field on the parameters of the plasma electron source and geometry optimization was shown in^19^. The magnetic field influenced the electrical breakdown characteristics in gas. The investigations that were performed in argon and nitrogen in both the crossed and parallel fields demonstrated the dependence of the electron yield in the magnetic field^20^.

### Effect of magnetic field in insulation systems of power devices

A magnetic field is generated by the current that flows through the conductors of transformers, power cables, gas-insulated systems, converters, etc. The globally observed trend toward electrically supplied devices is common also in the transportation segment. The influence of magnetic fields on PD in insulation systems of transportation power devices is presented in^21^. The measurements revealed enhanced PD intensity in the presence of a magnetic field that can be observed in a supply voltage frequency range of 20–400 Hz (which is typical for the transportation segment). The PD intensity was amplified in the above range at magnetic field induction 80 mT, even up to 50%. In power transformers, stray leakage magnetic fields of up to 700 mT may be present^6,22^; these could even be much higher during short-circuit currents, which would lead to winding deformations^23^. The effects of a magnetic field that is created by a load current on the partial discharge parameters in power cables and on the breakdown characteristics of impregnated paper insulation were shown in^24^. It was reported that the presence of an alternating magnetic field impacts the probability of electrical insulation breakdown and the Weibull distribution parameters. The morphology of the breakdown region is shown to be different under electric and electromagnetic field influence with scanning electron microscopy and energy dispersive spectroscopy. It was demonstrated that the performance of ozone production based on plasma reactors was improved when introducing a 0.4 T magnetic field in an interelectrode gap of 15 mm^25^, which led to higher discharge intensity. The production of magnetic field-enhanced DC corona ozone results in an extended current–voltage range and the observed stabilization of the discharges^26^. Similarly, enhancing cathode glow discharge intensity by a 650 mT magnetic field was observed in^27^, since the magnetic field was expected to increase the electron energy and promote electron collisional ionization. Electrical treeing in cross-linked polyethylene (XLPE) cable insulation subjected to a perpendicular magnetic field within a range of 350–550 mT was analyzed in^28^. It was shown that the tree morphology varied from the branch to bush types. The influence of a high gradient magnetic field on tree growth in epoxy resin was investigated in^29^. According to observations, the electrical tree inception and growth was accelerated in a gradient magnetic field. This result was explained by the rise in the conductivity of epoxy resin and dielectric loss tangents, as well as the drop in relative permittivity of the dielectric material subjected to a high magnetic field (up to 3 T). Simultaneously, it was shown that a strong magnetic field may generate more traps with shallower energy levels, decreasing the deep traps. As a consequence, the tree lengths were significantly longer after being subjected to a magnetic field than they were without a magnetic field^30^. According to observations magnetic field fostered the development of electrical trees at cryogenic temperatures; moreover, the effect on the length was greater than it was on the width^31^. Tree growth can also be impacted by a magnetic field while subjected to a repetitive train of pulses; this was demonstrated with silicone rubber in^32^. The effects of a magnetic field (0.4 ÷ 1.2 T) on the surface flashover of polyimide film for superconducting magnet insulation was described in^33^. The experimental results showed that a magnetic field drifting into a surface may block secondary electron emission, leading to an increase in the flashover voltage. It was also reported that the breakdown voltage depended on the inclination angle between the magnetic field vector and the surface of the dielectric material^34^. The investigations of magnetic field impact on corona discharges in oil were presented in^35^ using fluorescent and UHF detection. It was observed that the dominant frequency of the UHF signal obtained under AC voltage shifted towards a lower frequency (0.6 GHz) under the influence of the magnetic field (85 mT).

This paper is focused on the original measurement approach, detection and quantitative visualization of the impact of a magnetic field on the PD dynamics in air. The presented results are based on both simulations and measurements carried out in dedicated setup. The quantitative comparison of PDs evolution in the magnetic field is a novel aspect shown in this paper. Important difference is, that previous published works have been performed in strong magnetic field. In the experiments presented in this paper, a quite weak magnetic field was set in order to detect PD behavior at the bottom-line field level in power devices. Actually, two distinctive cases were considered: the first one refers to PDs in gaseous (air) voids, and the second to corona discharges.

### Partial discharges in magnetic field

**Figure 1:**
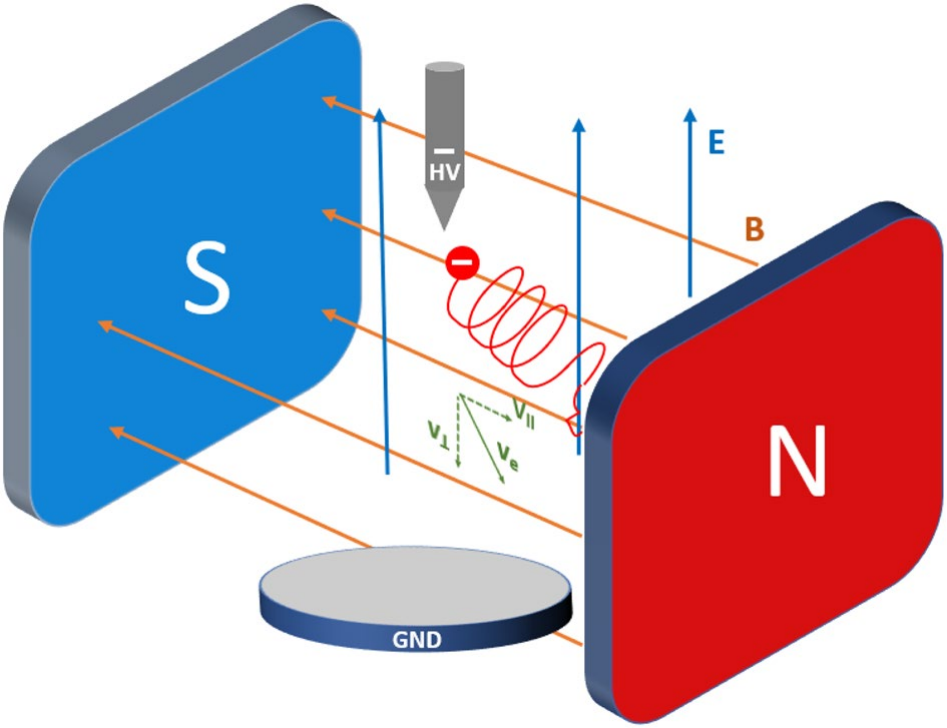
Visualization of particle trajectory in crossed magnetic *B* and electric *E* fields.

The theoretical part about discharges and electron behavior in magnetic field is highlighted for general information because conventionally in the PD discipline usually only the electric field is considered. Thus, such an introduction will also underline the magnetic aspects. The partial discharges that appear in a strong electric field are described by the inception and propagation of charge carriers and are detected as current pulses. These streamers usually propagate along the electric field lines^36^. The path may also be influenced by dielectric surfaces^37–39^ or barriers. Since HV insulation is exposed to magnetic fields in all current-carrying devices such as cables and power transformers, the electrical exposure refers to the high voltage; however, the magnetic field is related to the load. In this way, partial discharge behavior is potentially modulated by the current flow. The magnetic field impacts the pathway and dynamics of the particles in the gas, thus forming the discharge trajectory. The visualization of streamer propagation in crossed magnetic *B* and electric fields *E* is presented in Fig. 1. In addition to the orientation of the *B* and *E* fields, the initial direction of charged particle velocity vector *v* impacts the drift propagation. Depending on the initial particle velocity, this can assume various shapes (cycloidal, trochoidal, spiral, helical, etc.).

The focus of this paper is at this stage on the charge trajectory in air under a magnetic field, not taking into account neither collisions in gas nor the bulk and surface effects in the dielectric material. Thus, the theoretical part and the simulations shown in the next paragraph refer to the behavior of particles in a vacuum, without collisions. The charges move in the superimposed magnetic and electric fields (***B*** and ***E***) that are affected by Lorentz force *F*_*L*_:

1 $${{\varvec{F}}}_{L}=q({\varvec{E}}+{\varvec{v}} \times {\varvec{B}})$$

where *q* is the particle charge, *m* – the particle mass, and *v* – the particle velocity.

A particle with a charge of *q* performs a circular motion around the vector of magnetic field induction *B* with a frequency of *f*_*B*_:

2 $${f}_{B}=\frac{q}{2\pi m}B$$

Frequency *f*_*B*_ is independent of the particle velocity; thus, fast-moving particles move on trajectories with higher radii than slower ones. For example, *f*_*B*_ is equal to 2.8 GHz for electrons in a 100-mT field.

Two components of the electron velocity vector are shown in Fig. 1: parallel to the magnetic field vector, and perpendicular to it. Electron transport velocity *v*_*e*_ at accelerating voltage *U* can be estimated in a simplified form from the following:

3 $${v}_{e}=\sqrt{\frac{2eU}{{m}_{e}}}$$

where *m*_*e*_ denotes electron mass 9.1 × 10^–31^ kg.

Radius *r*_*e*_ of the electron circular pathway is:

4 $${r}_{e}=\frac{{v}_{e}}{2\pi {f}_{B}}$$

thus, the radius of electron cycling will be equal to *r*_*e* _= 3.4 mm in a magnetic field with *B* = 100 mT and accelerating voltage *U* = 10 kV.

Simultaneously, the magnetic moment of the particle presses around the external magnetic field vector with Larmor frequency *f*_*L*_:

5 $${f}_{L}=\frac{\gamma }{2\pi }B$$

where γ is a gyromagnetic ratio (for electron 1760 × 10^8^ Hz/T and proton 2.67 × 10^8^ Hz/T). The resulting Larmor frequency *f*_*L*_ for the electron and proton at 100 mT yields 2.72 GHz and 4.26 MHz, respectively^40^. The latter is utilized in nuclear magnetic resonance (NMR).

The drift equation for the particles in the crossed electric and magnetic fields is as follows (taking collision time *τ* that is defined by^1^ into account):

6 $${m}_{e}\frac{d{v}_{e}}{dt}=q\left({\varvec{E}}+{\varvec{v}} \times {\varvec{B}}\right)-\frac{{m}_{e}{v}_{e}}{\tau }$$

where electron collision time *τ* can be expressed as follows:

7 $$\tau =\frac{{\lambda }_{e}}{{v}_{t}}={\lambda }_{e}\sqrt{\frac{{m}_{e}}{k{T}_{e}}}$$

This denotes the *λ*_*e*_ electron mean free path, *v*_*t*_ electron thermal velocity, and *k* Boltzmann constant, *T*_*e*_ electron temperature. The electron collision frequency is proportional to the gas density and is at a level of 3 × 10^12^ Hz^40^. The particle trajectory is along the cycloidal orbits in a direction that is perpendicular to the magnetic and electric fields. Those charges that move in the crossed magnetic and electric fields are scattered in different ways: elastic (when the direction is altered but the energy remains constant) or nonelastic (in case of atom ionization by collision)^1^. It is assumed that the pathway that connects consecutive ionization steps in the crossed *E* and *B* fields determines the streamer path.

For assessing the influence of the magnetic field on the charge movement in the electric field, a relative comparison of the electric and magnetic forces (*F*_*E*_ and *F*_*B*_) that acted on the electron was performed:

8 $$\frac{{\mathrm{F}}_{E}}{{F}_{B}}=\frac{1}{{v}_{e}}\cdot \frac{E}{B}$$

where *E* is the electric field strength, *B*—induction of the magnetic field, and *v*_*e*_—electron velocity.

The comparison can be obtained by taking the values of the electron drift velocity that is used in gas discharge physics (which, for electric field *E* = 10 kV/cm at pressure *p* = 1013 hPa and at room temperature T = 293 K is within the ranges of *v*_*e*_ = 2.9 × 10^6^ cm/s)^41^, *v*_*e*_ = 6 × 10^6^ cm/s^36^, or according to the equation in dry air^42^:

9 $${v}_{e}=3.4\cdot {10}^{7}\frac{E}{p}+5\cdot {10}^{6}$$

where *v*_*e*_ is in [cm/s], *E* in [V/cm], *p* in [Pa], and the electron velocity is equal to *v*_*e*_ = 8.3 × 10^6 cm^/s. For the above three electron drift velocities, at induction of magnetic field equal to *B* = 80 mT, ratio *F*_*E*_*/F*_*B*_ yields 448, 216, and 156, respectively, according to Eq. (8); this indicates the rough and simplified level of the contribution of a magnetic force. In the case of ions, the ratios will be bigger since they have hundreds-of-times-smaller velocities; this indicates that the contribution will be less significant.

In the experiment, the applied voltage (frequency 50 Hz) controlled the electric field and the magnetic field oriented perpendicularly remained constant. While analyzing the trajectories of individual discharges, both fields can be treated as constant since the streamer propagation time is within a range of nanoseconds.

### Simulation of charged particle trajectory in crossed electric and magnetic fields

**Figure 2:**
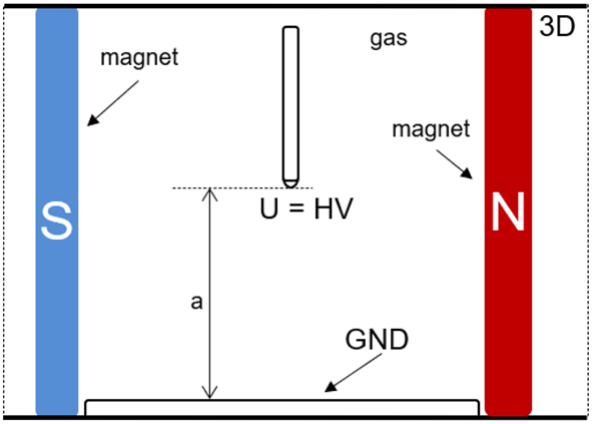
View of the 3D configuration of simulation domain (cross-section).

The simulations were used to investigate the trajectories of the particles in the cross-coupled magnetic and electric fields. The intension of the simulation was to study a hypothetical pathway subjected to the superposition of both magnetic and electric forces, without taking into account interactions of particles such as collisions. In that sense, the theoretical part and simulations shown in this paragraph refer in fact to propagation similar to vacuum conditions. For collisions in air under normal pressure, the electron length path is very short, and the whole mechanism will differ. However, the intension was in a first step to visualize the effect of electric and magnetic field interplay, mimicking the trajectory, which is, in fact related to vacuum. In reality, the discharge is moving along the electric field lines due to the ongoing ionization process; thus, the key point was to investigate the trajectory due to the presence of the magnetic field. It is in fact a channel consisting of electrons and ions, which will be impacted in a more macroscopic view by a superimposed magnetic field. In that sense, the simulation has a more qualitative character to highlight this mechanism. In order to interpret the potential root cause of the elevated number of partial discharges in a magnetic field, a simulation of the corona discharge traces in the point-plane configuration was performed. Because the individual propagation time of a discharge is within nanoseconds, in the simulations the DC condition was taken, reflecting an instantaneous slot on the sinusoidal waveform. The motion Eq. (1) (the Lorentz force) of a charged particle was solved in 3D geometry in both electric and magnetic fields. The numerical simulations were performed in the COMSOL Multiphysics framework^43^. The simulation sequence consists of two consecutive elements: in the first step, the magnetic and electric fields are computed. Afterwards, charge particle tracing is carried out in the subsequent step, in the superimposed magnetic and electric fields reflecting the motion equation and resulting forces. The obtained trajectories can follow cycloidal or helical pathways in both fields. Therefore 3D simulations are suggested, particularly when non-homogeneity or gradients along the axes in electric and magnetic fields are also present. View of the 3D configuration of simulation domain (cross-section) with the variable air gap that was used for the simulation is presented in Fig. 2. The tetrahedral elements were used in meshing. The HV electrode with a tip that has a curvature of 60 μm was placed at distance *a* from the plane radial ground electrode.

**Figure 3:**
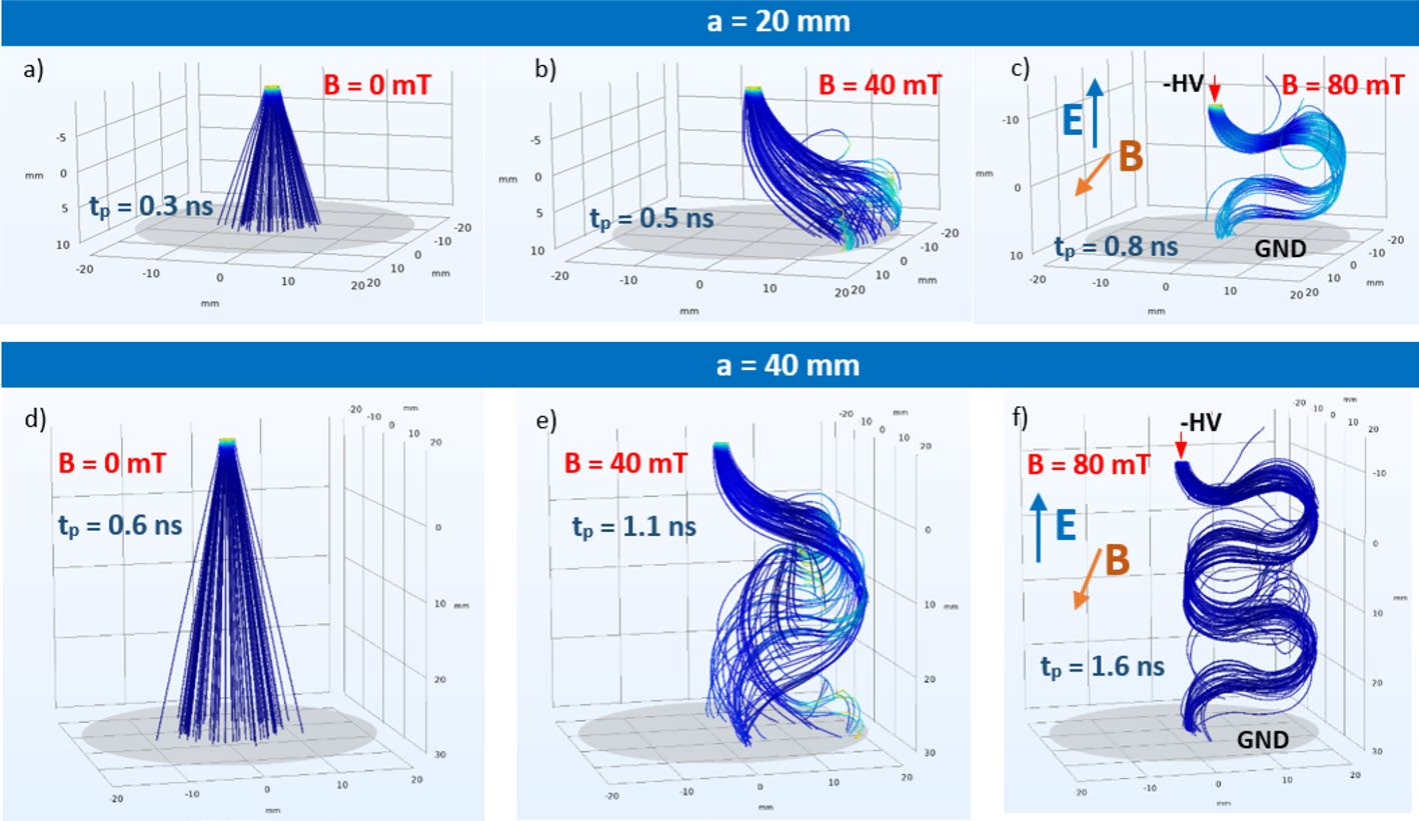
Comparison of electron trajectories in crossed electric and magnetic fields for interelectrode gap distance *a* = 20 mm (upper row) and *a* = 40 mm (lower row) at HV voltage *U* = − 10 kV for magnetic field induction: (**a**) *B* = 0 mT; (**b**) *B* = 40 mT; (**c**) *B* = 80 mT; (**d**) *B* = 0 mT; (**e**) *B* = 40 mT; (**f**) *B* = 80 mT. Orientation of *E*, *B* fields and electrode description in Graphs (**c**) and (**f**).

The set of boundary conditions for the simulations indicated in the graph were adopted: the needle electrode was at the HV potential, whereas the plane electrode was at the ground potential. The freeze option was activated when the particle was in contact with a wall. The source of the particles was attached to the HV electrode. According to assumed simplification, streamers that originated from the ionization in the airgap are not reflected; however, it seems to be a good approximation for the purpose of these simulations. The presented calculations were executed with DC voltage at a HV electrode in a range of − 8 to − 20 kV. The standard conditions (STP) (i.e., a pressure of 0.1 MPa and temperature of 300 K) were taken in the simulations. The HV tip was used as a particle beam, releasing 1000 particles from a beam source in a simulation sequence. In the presented examples, at this stage of simulations, the electrons were used in the particle-tracing comparison. As an initial condition, the mean kinetic energy of 5 keV was defined with Gaussian standard deviation σ being equal to 0.1 eV. The trajectory of the particle was rendered as a tube in the graphical visualization. The simulations were executed for two values of gap distances (*a* = 20 mm and *a* = 40 mm between the HV electrode and the ground). Comparisons of the trajectories for both of the interelectrode distances at HV voltage of *U* = − 10 kV are presented in Fig. 3 for the magnetic field-free instance and two other scenarios with magnetic inductions equal to *B* = 40 mT and *B* = 80 mT. Without presence of the magnetic field (*B* = 0 mT—Fig. 3a, d), the electrons move along straight lines from the beam source at the cone-shaped tip of the HV electrode. Applying a perpendicular magnetic field with an induction of *B* = 40 mT caused the twisting and deflection of the trajectory (Fig. 3b, e) following the Lorentz force. Amplification of the magnetic field induction caused a circling of the beam around the magnetic field axis with a simultaneous drift due to an electric field toward the ground electrode. It is notable influence of the magnetic field on the propagation time of the charged particle. For a gap distance of *a* = 20 mm, the electrons reached the ground level in the field-free case within 0.3 ns, whereas the presence of magnetic fields with induction *B* = 40 mT and *B* = 80 mT yielded 0.5 and 0.8 ns, respectively. In addition, the presence of a magnetic field led to a more focused and localized spot (e.g., Fig. 3c, f) that was not dispersed as in the field-free scenario (Fig. 3a, d).

It was observed that the elongated trajectory due to the magnetic field deflection led to a longer residence and propagation time *t*_*p*_ of the whole pathway between the HV and ground electrodes. A comparison of these values is presented in Table 1. In this simulation, no collision nor ionization effects have been included.

**Table 1:** Electron beam propagation time *t*_*p*_ [ns] in point-plane setup in E × B.

*t*_*p*_ [ns]	Magnetic field *B* [mT]		
Distance *a* [mm]	0	40	80
20	0.3	0.5	0.8
40	0.6	1.1	1.6

**Figure 4:**
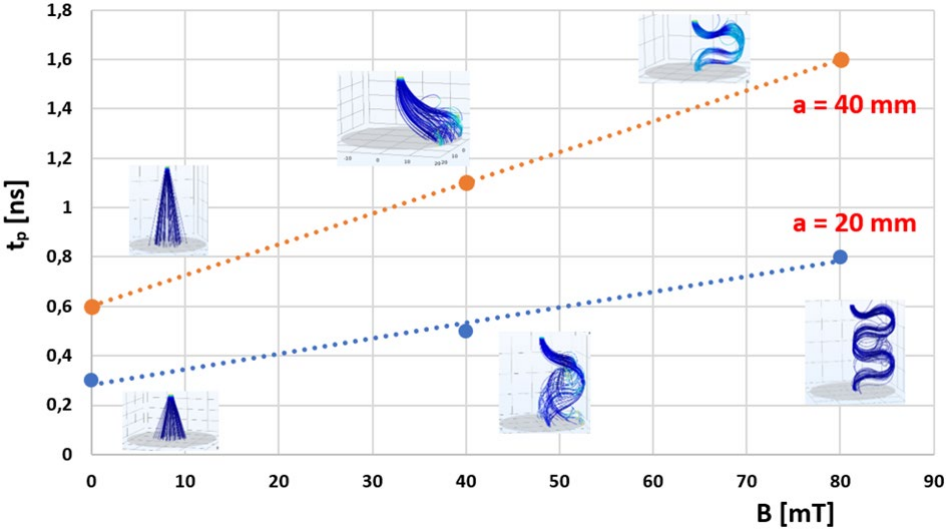
Electron beam propagation time *t*_*p*_ as function of background magnetic field *B* in point-plane configuration, *a*—interelectrode distance.

Electron beam propagation time *t*_*p*_ as a function of the background magnetic field in the point-plane configuration is shown in Fig. 4. Interelectrode distance *a* is a parameter. The relationship is linear in the defined range of magnetic induction *B*, and the slope changes depending on the value of *a*.

**Figure 5:**
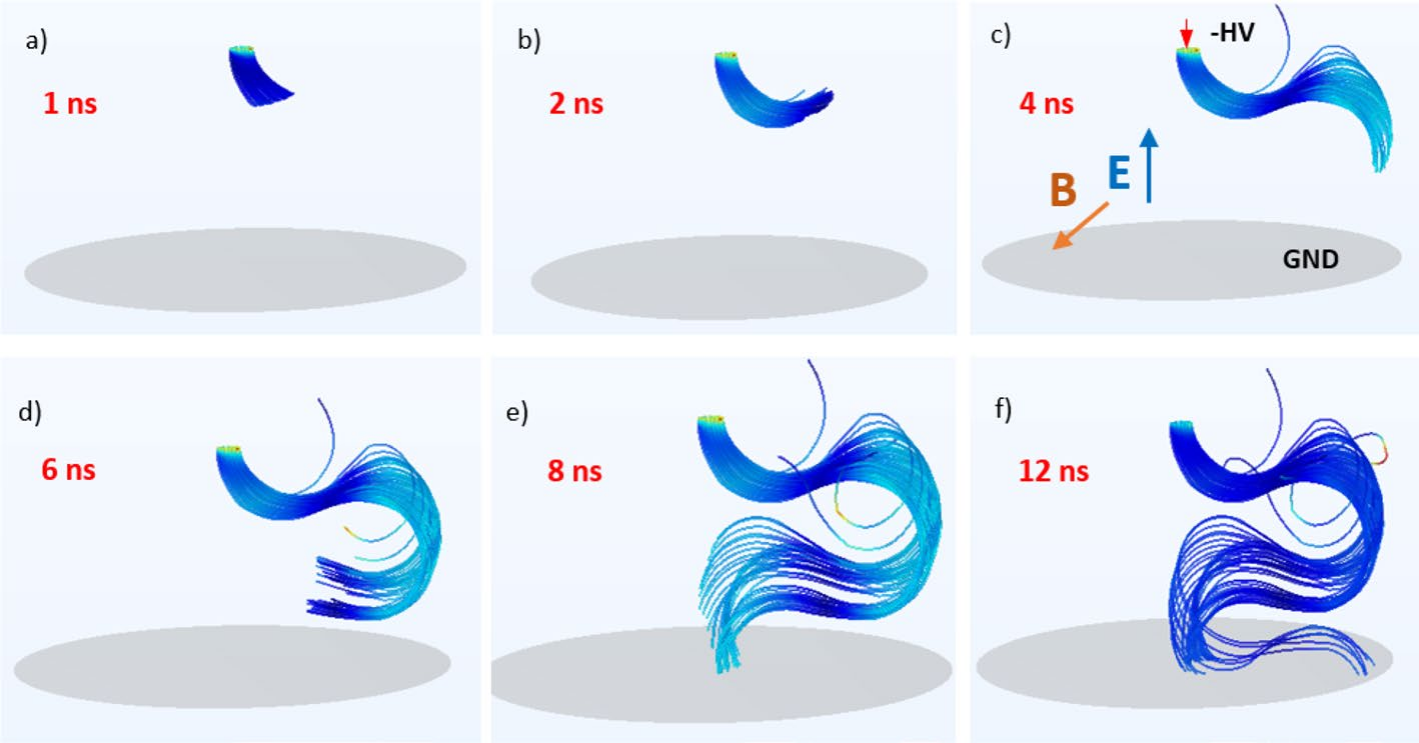
Frame sequence of propagation trajectory evolution over time for induction *B* = 80 mT, distance *a* = 20 mm, and *U* = − 8 kV at time stamps: (**a**) 1 ns; (**b**) 2 ns; (**c**) 4 ns; (**d**) 6 ns; (**e**) 8 ns; (**f**) 12 ns—orientation of *E*, *B* fields and electrode description in Graph (**c**).

A frame sequence that shows the development of the propagation pathway over time for magnetic field induction *B* = 80 mT and HV voltage *U* = − 8 kV is shown in Fig. 5. The trajectory swirl can already be observed at the exit of the beam source (Fig. 5a), following side deflection (Fig. 5c) and almost full turn (Fig. 5e).

A certain composition of the ratio of the electric and magnetic forces may even lead to a wrapping of the original trajectory, leading to the attenuation or even stoppage of the streamer propagation.

**Figure 6:**
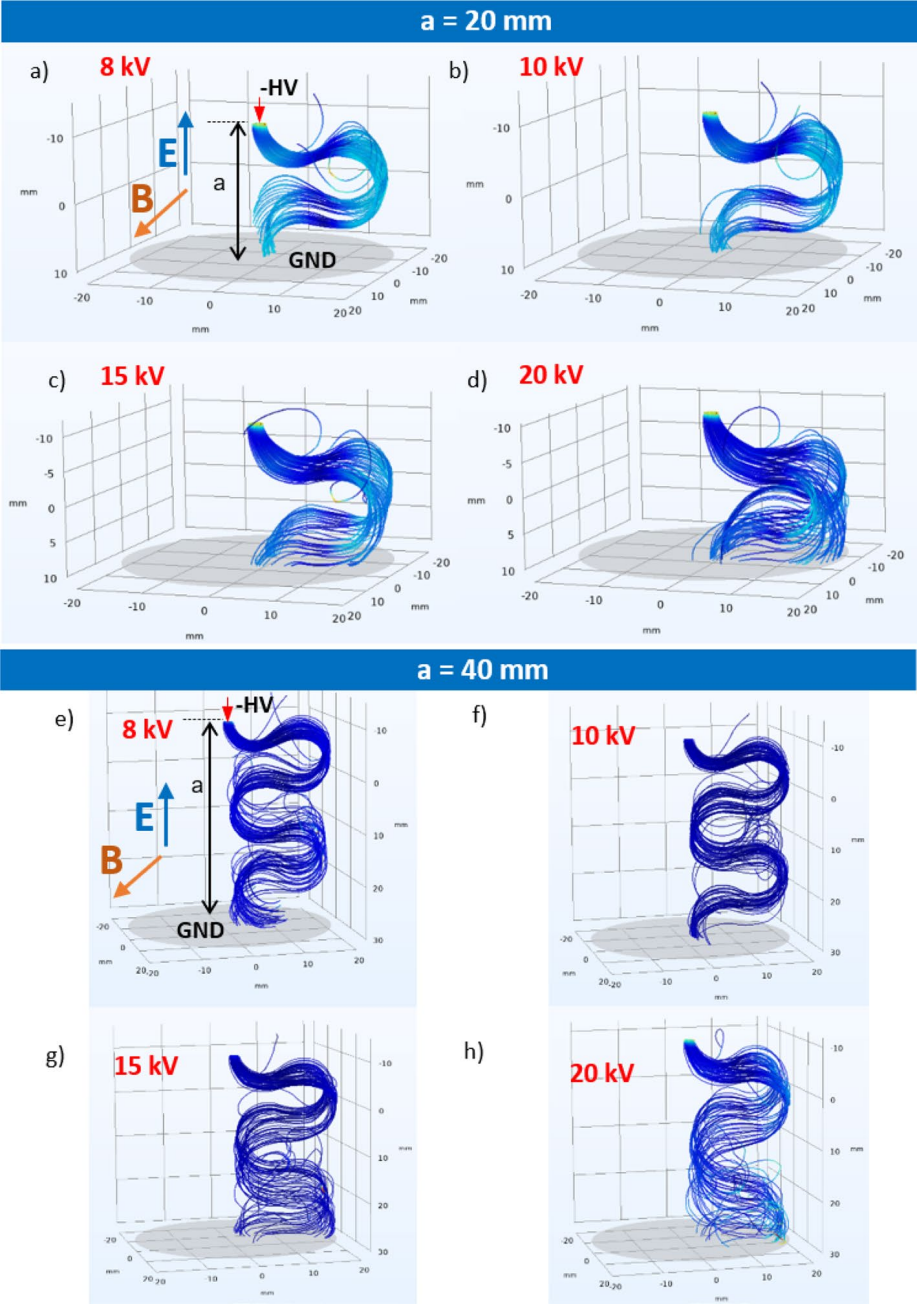
Electron beam trajectories in crossed electric and magnetic fields for interelectrode gap distance *a* = 20 mm (**a–d**) and *a* = 40 mm (**e–h**) at magnetic field *B* = 80 mT for applied voltages: (**a**) − 8 kV; (**b**) − 10 kV; (**c**) − 15 kV; (**d**) − 20 kV; (**e**) − 8 kV; (**f**) − 10 kV; (**g**) − 15 kV; (**h**) − 20 kV.

When controlled by the electric field strength, the acceleration voltage also influences the shape of the charged particle trajectory. This dependence for an electron in a magnetic field with *B* = 80 mT in an corona arrangement with gap distances of *a* = 20 mm and *a* = 40 mm is visualized in Fig. 6 for applied voltages within a range of − 8 to − 20 kV. A comparison of the electron paths in the shorter and longer interelectrode distances reveals a duplication of the core pattern at the same voltage level (e.g., Fig. 6a and e). The aim of the simulations was to compare the beam trajectory modulation by a perpendicular magnetic field.

### Experimental setup and instrumentation

**Figure 7:**
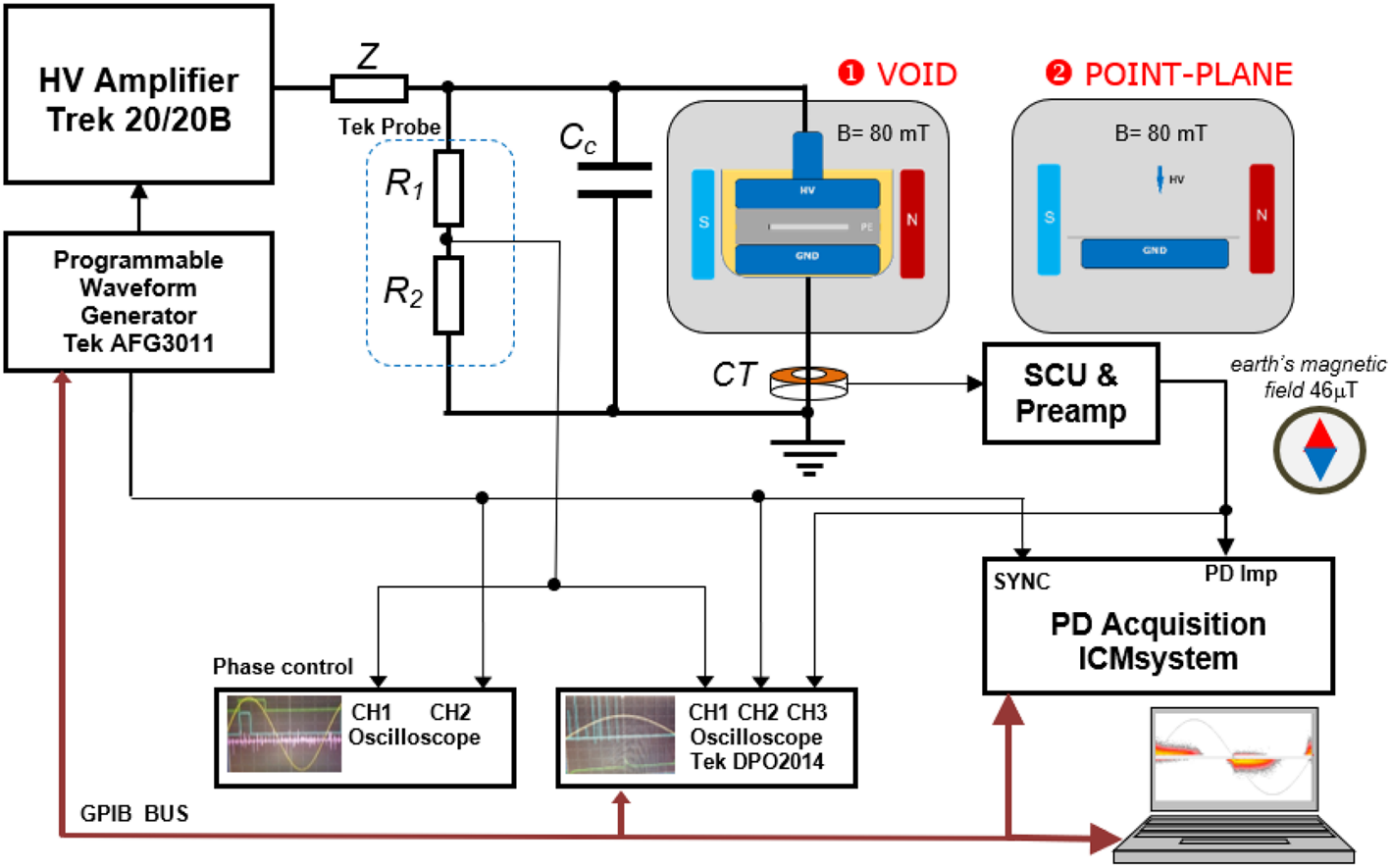
Instrumentation for partial discharge measurement in crossed electric and magnetic fields: **(**1) void in PE specimen; (2) point-plain corona arrangement.

The measurements were performed in the original setup which allowed for sensitive detection in the presence of weak magnetic field, since conventionally PD measurement are carried out only in the electric field^21^. The presented experiments were performed in the crossed magnetic and electric field setup; the experimental arrangement is presented in Fig. 7. Two types of investigations were carried out: first—on an air void that was embedded in polyethylene (PE), and second—in a point-plane configuration with a PE barrier placed on the ground electrode. All of the elements within the experimental setup that are exposed to the magnetic field should be made of non-magnetic material (especially the electrodes and connections). The static magnetic field in this experimental arrangement was provided by two permanent neodymium magnets that were located on both sides of the electrodes at a distance 70 mm, creating a quasi-uniform field distribution in the interspace. The magnetic induction in the middle of the gap was 80 mT. The magnets were placed and removed manually in the experimental setup with high precision. The magnets were fixed with wooden construction to provide a stable arrangement. The magnetic field induction was measured using an SMS 102 m equipped with a Hall sensor. For information, the direction of Earth’s north pole was perpendicular to the static field and is denoted in Fig. 7 by a symbolic compass.

**Figure 8:**
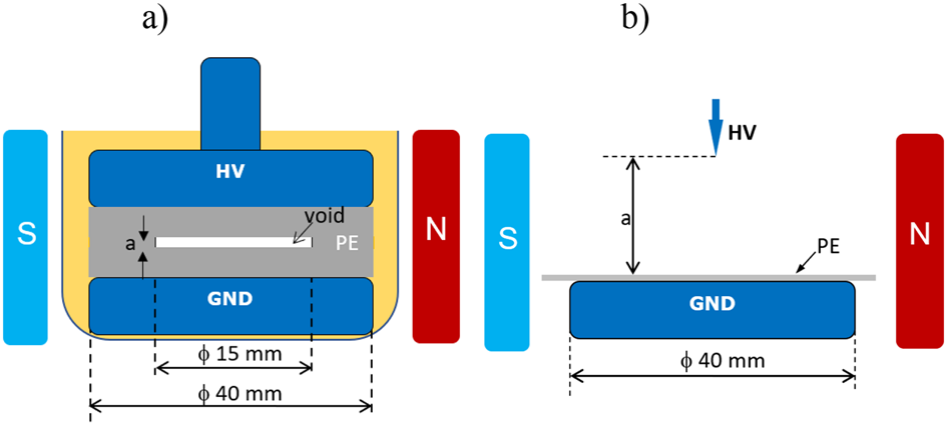
Specimen geometry: (**a**) embedded void in PE; (**b**) corona point-plane configuration.

The geometry of the specimen that contained the void is presented in Fig. 8a. The void specimen had dimensions of 50 × 50 mm and a thickness of 3 mm. The samples with internal voids that had a diameter of 15 mm, and two variants of thickness (*a*_*1*_ = 240 μm and *a*_*2*_ = 1 mm) were used in the tests. The electric permeability ε_r_ of the PE material was equal to 2.2. The HV and ground electrodes had a diameter of 40 mm are were made of polished aluminum. In order to avoid surface discharges, the whole setup with a sample was immersed in oil.

The corona point-plane geometry is shown in Fig. 8b. The HV needle electrode was made of copper, and the grounded electrode with a diameter of 40 mm was made of aluminum. The tip of the point HV electrode had a spherical radius of *r* = 60 μm. The PE dielectric barrier with dimensions of 60 × 60 mm was placed on the ground electrode. Interelectrode gap *a* was changed within a range of 20 to 60 mm. The HV sinusoidal waveform was delivered from an amplifier (model Trek 20/20B) that was controlled by a function generator (Tektronix Model AFG 3011). Limiting resistor *Z* was placed in the high-voltage path at the output of the HV source. For closing the high-frequency partial discharge loop, coupling capacitor *C*_*c*_ was placed in a parallel branch of the specimen. The PD were acquired in the wideband phase-resolved mode (PRPD) using an ICM-acquisition system by Power Diagnostix. The unit was attached to a control computer by means of a GPIB interface. The detection of the PDs was performed using a wideband current transformer CT that was terminated at 50 Ω. The presented experiments were executed at room temperature (21 °C) with a humidity level of 24% and an atmospheric pressure of ca. 0.1 MPa.

## Results and discussion

The interesting observation that is reported in this paper refers to the modulation; i.e., the amplification or attenuation of PD intensity by a superimposed magnetic field. In order to investigate the influence of a magnetic field on partial discharge dynamics, two types of experiments were conducted: first—in a gaseous (air) void; and second—in a point-plane corona configuration in air with a dielectric barrier. The discharge dynamics was indicated and visualized in the performed experiments by the number of discharges that were recorded within a predefined time interval and is referred to as intensity in this paper. The experiments were performed in a static magnetic field with an induction of 80 mT. This relatively low field was applied in order to detect PD behavior in a field range that can possibly occur in everyday power devices. Regardless, this field is three orders of magnitude higher than Earth’s magnetic field (e.g., in Krakow, this is equal to ca. 46 μT).

### Gaseous inclusion

**Figure 9:**
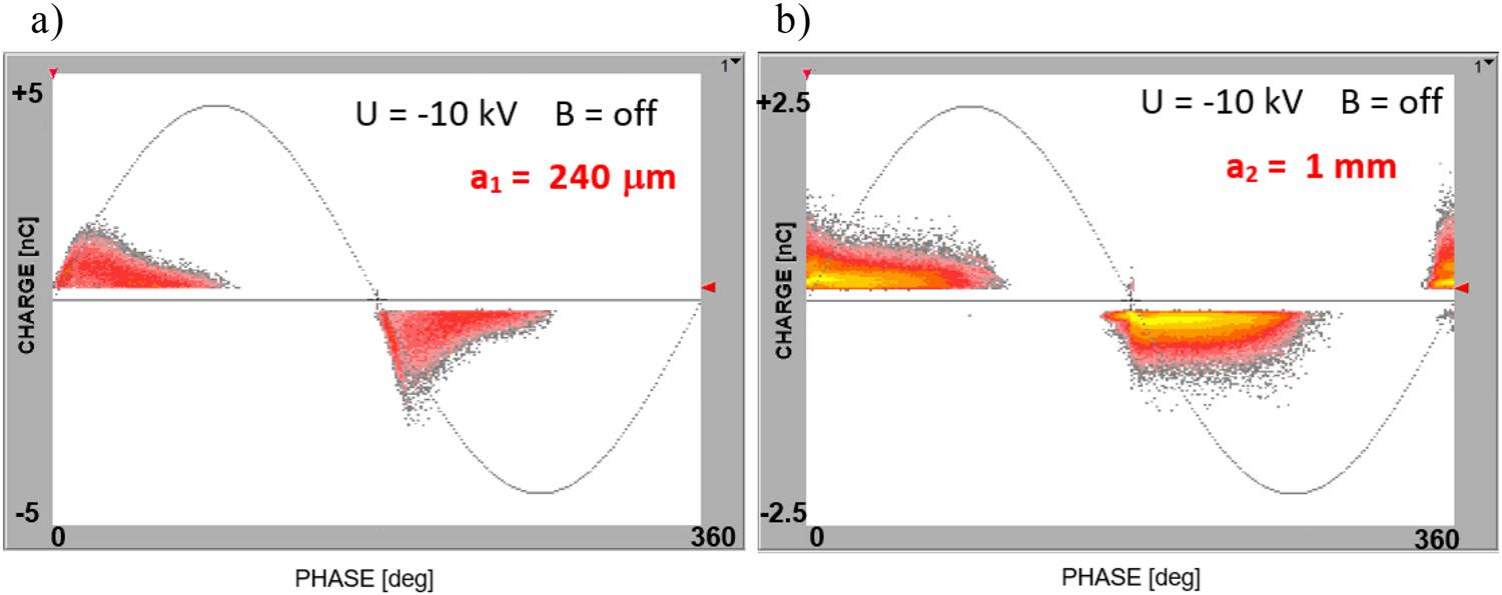
PD patterns recorded at 10 kV (B = off) for sample with embedded voids with thicknesses of (**a**) 240 μm and (**b**) 1 mm.

The partial discharge measurements were performed in the experimental arrangement that is shown in Fig. 7 as well as in a PE specimen that contained an air void (shown in Fig. 8a). The void’s diameter was 15 mm, and specimens with two thicknesses were investigated (*a*_*1* _= 250 μm, and *a*_*2* _= 1 mm). The PD inception voltages for these samples were 8 and 7.6 kV, respectively. With the presence of magnetic field both PD inception voltages were slightly lower, yielding 7.5 and 7.2 kV. The inception voltages are quite similar, and this effect refers to the breakdown voltage in the air at small distances. Namely, the common value of electric field withstand for few millimeters interspaced cavity at normal pressure is 3 kV/mm, whereas for submillimeter distance it goes up to 5 kV/mm, and further to 9 kV/mm for tiny voids 0.01 mm thick^10^. The PRPD patterns that were recorded at 10 kV for the specimen with thickness *a*_*1*_ are shown in Fig. 9a; for thickness *a*_*2*_ in Fig. 9b, the graph reflects typical images that corresponded to the presence of a gaseous inclusion in the dielectric material^10^.

**Figure 10:**
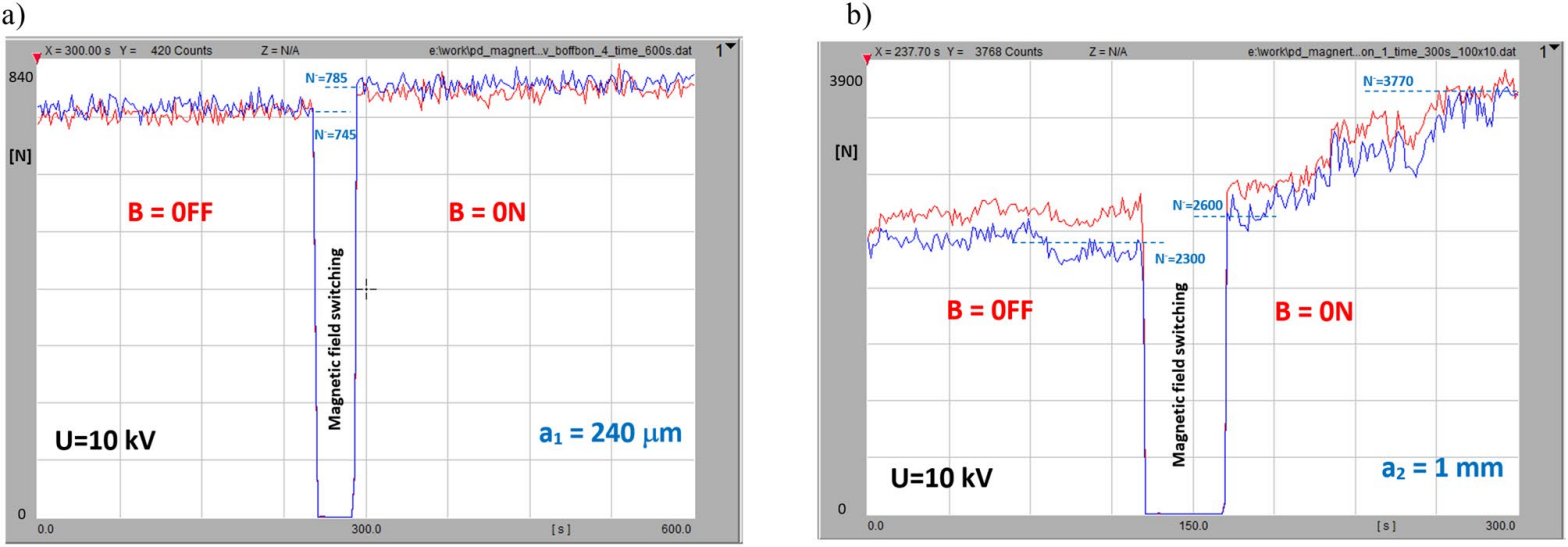
Time sequence B = off/B = on of impact of magnetic field on PD intensity in PE void at 10 kV with following thicknesses: (**a**) *a*_*1*_ = 240 μm; (**b**) *a*_*2*_ = 1 mm. The number *N* of negative and positive PD pulses is denoted in red and blue, respectively.

For revealing the impact of the magnetic field, the measurement scenario was split into two phases (shown in Fig. 10): first—magnetic field-free (B = off), followed by a switching gap lasting for about several seconds; second—with active magnetic field (B = on) with an induction of 80 mT. The two traces denote the numbers of negative and positive partial discharge pulses (represented in blue and red, respectively). The observed small difference between the number of negative and positive PD pulses may be attributed to the large surface void wall of the specimen and related nonuniformities (including surface profile and micro ripples) also to the surface conditions, since the void was manufactured from the PE layers. Thus this difference results from a tiny asymmetry in the experimental setup. As shown in Fig. 10a, switching the magnetic field on manifested an increase in the waveform level for a void with a thickness of 240 μm in the partial discharge time acquisition of the B = off/on sequence. For the thicker void (*a*_*2* _= 1 mm) in the PE, the PD intensity, denoted by number of discharges *N*, rapidly grew after switching on a magnetic field with an induction of 80 mT (as shown in the PD time sequence in Fig. 10b). The revealed rise of the PD intensity while switching on the magnetic field may refer to the elongated pathway of both the ions and electrons, which were caused by the coexistence of the crossed magnetic and electric fields.

**Figure 11:**
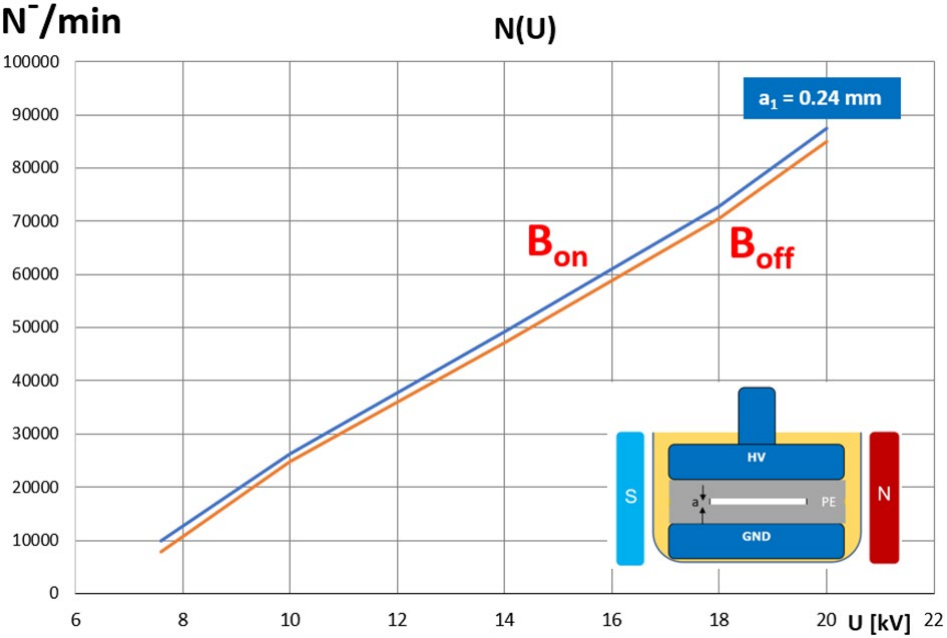
Relationship of number of negative polarity PD pulses versus applied voltage for both magnetic field-free and magnetic field-present measurements (PE specimen, void thickness *a*_*1*_ = 240 μm).

The number of PD pulses of both polarities grew for a thinner inclusion (*a*_*1* _= 240 μm) from 745 (the average of both polarities) at the steady state to 785 (just after the magnetic field transition). For the thicker void (*a*_*2*_ = 1 mm—Fig. 10b), the field-free level yielded 2300 and increased at a constant rate up to 3770 (both polarities) within 140 s. It is worth noticing that that, in the field-free stadium, the number of positive and negative PD pulses was slightly different, whereas these values were more convergent in the magnetic field. The relationship between the number of negative polarity (N^-^) PD pulses versus the applied voltages within a range of 8 to 20 kV for both the magnetic field-free and magnetic field-present measurements is shown in Fig. 11.

The plot reveals a high degree of linearity, confirming the slightly higher PD intensity throughout the entire voltage range. Since the PD pattern was symmetrical, the graph for the positive pulses is very similar. Since the magnetic field influenced the flight path of the charged particles (i.e., the electrons and ions), there is a certain critical inclusion geometry where this effect is substantial. In the investigated void geometry and at a magnetic field induction of 80 mT, this effect was not detected below the thickness of ca. 100 μm (even the attenuation of the PD number was noticed). Thus, comparing these cases (i.e., field-free, and the presence of a magnetic field), a significant influence was observed (especially on the PD intensity). The effect of a magnetic field on the PDs was more predominant for the thicker void. The non-uniformity of the magnetic field may have additionally influenced the charge trajectory locally. The number of collisions between the electrons, ions, and gas molecules was enhanced by a magnetic field along the elongated trajectory, including amplification of electron energy due to acceleration in the magnetic field; these were the root causes of the investigated phenomena.

### Corona in air with dielectric barrier

**Figure 12:**
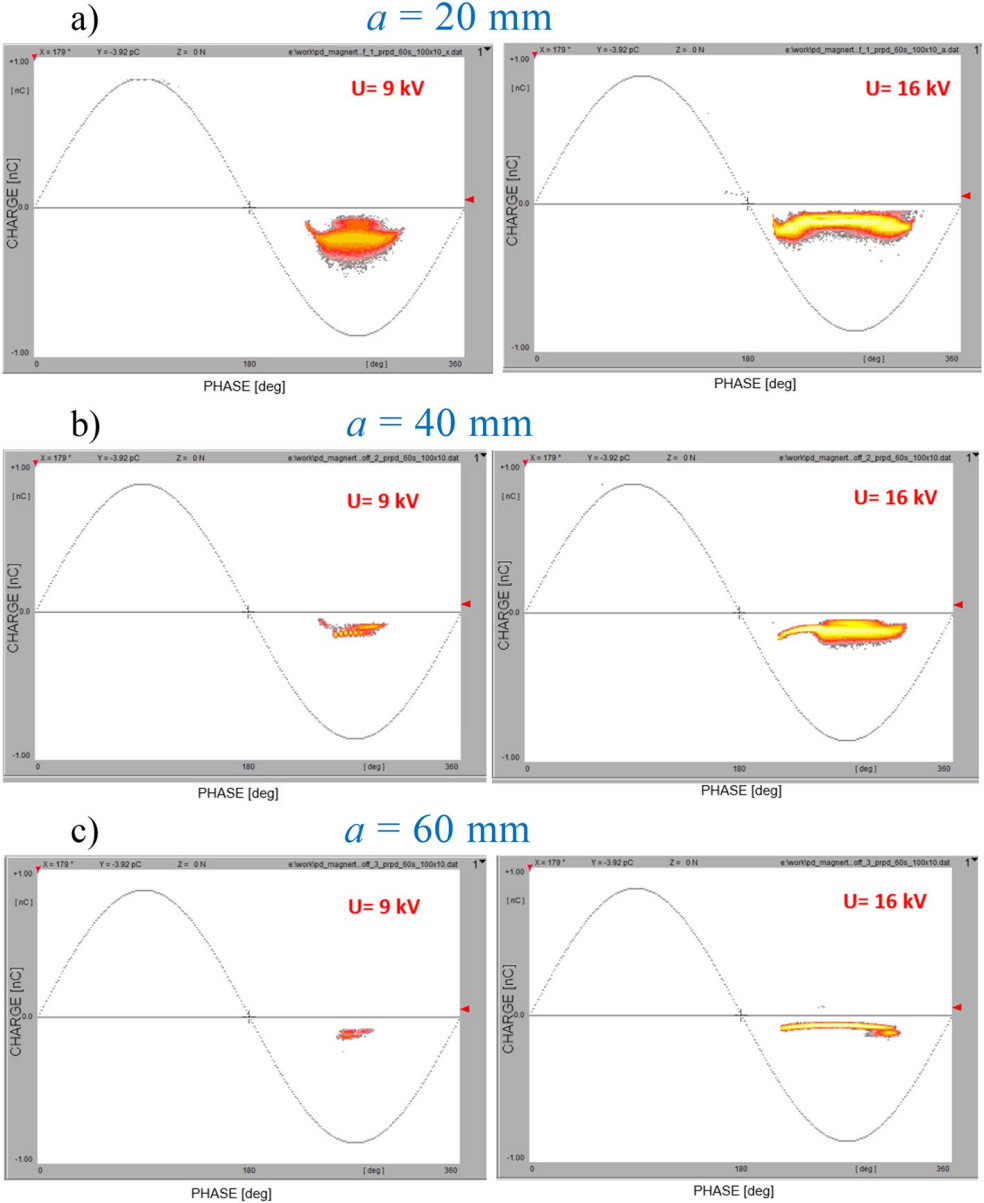
PD patterns recorded (B = on) for two extreme voltage values (9 kV—left column, and 16 kV—right column) and for three interelectrode distances: (**a**) *a* = 20 mm, (**b**) *a* = 40 mm, (**c**) *a* = 60 mm.

The second set of experiments was performed in a point-plane corona configuration in air with a dielectric barrier (PE) placed on the ground electrode^44,45^; the measurement setup is shown in Fig. 7. The parameter in these measurements was distance *a* between the tip of the point electrode and the ground (assuming values of 20, 40, and 60 mm). A constant magnetic field with induction *B* equal to 80 mT was applied in the direction that was perpendicular to the point-plane arrangement (thus, to the electric field). The PD patterns that were recorded for above-mentioned three interelectrode distances and two extreme voltage values (9 and 16 kV) are shown in Fig. 12. The lower value is close to inception, whereas the upper one refers to the saturation level.

**Figure 13:**
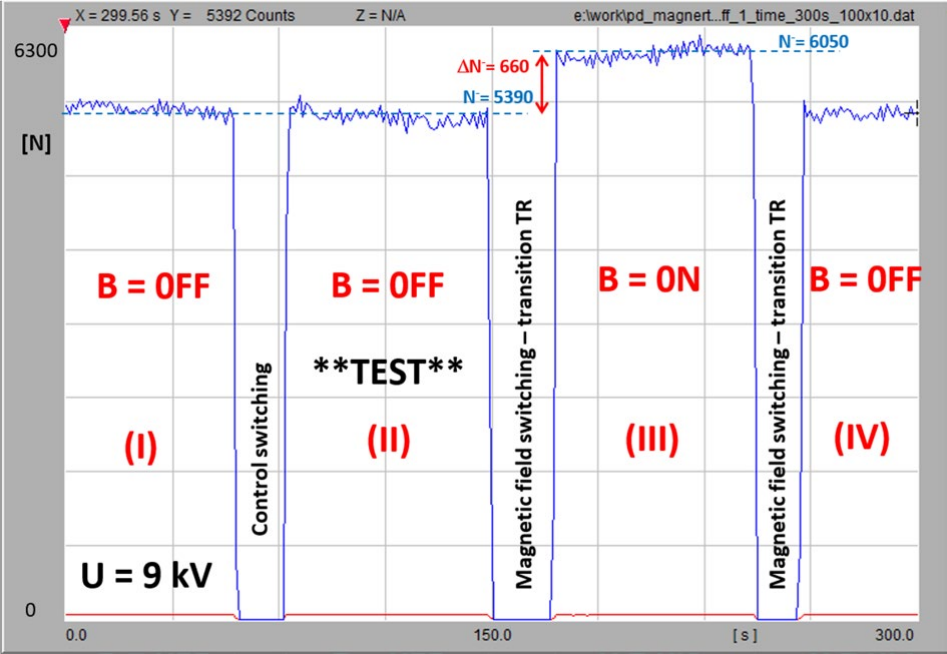
PD-intensity diagram of test-switching sequence: (I) initial magnetic field-free state; (TR) test turn-off switching; (II) PD recording at B = off; (TR) transition for switching on magnetic field; (III) presence of magnetic field; (TR) transition to field-free state; (IV) magnetic field off.

**Figure 14:**
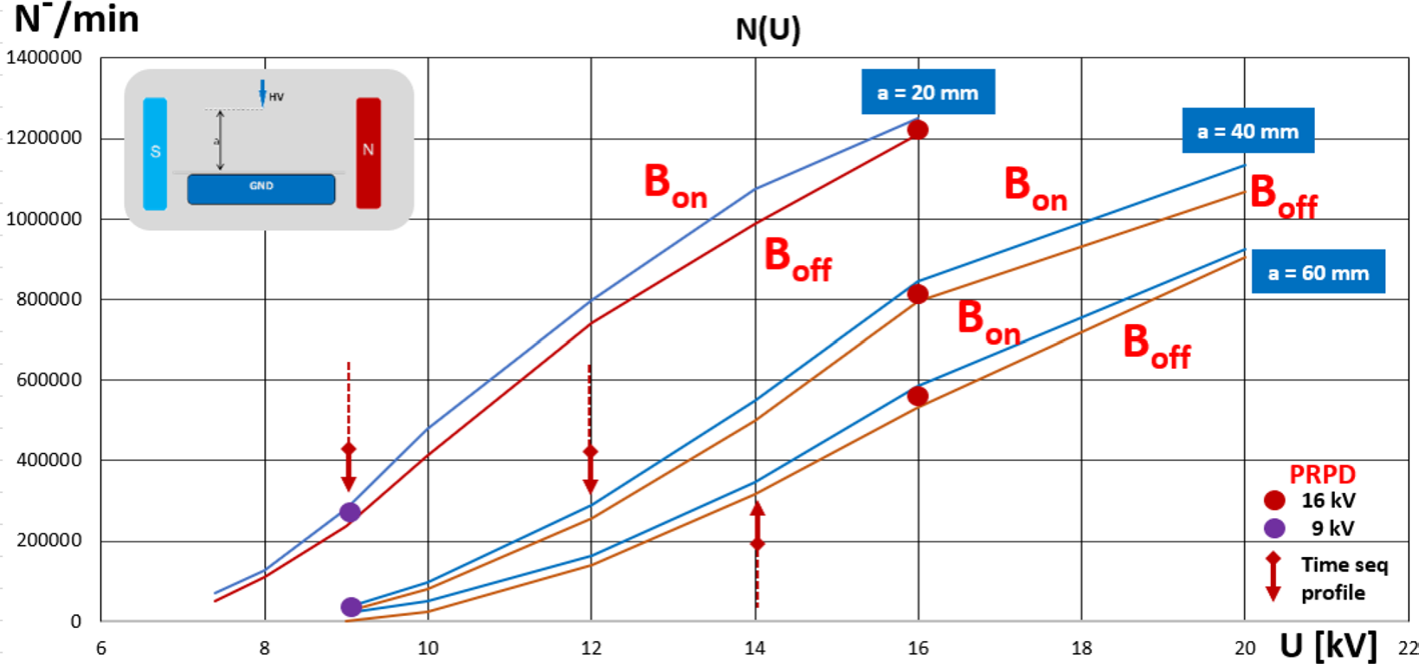
Relationship of number of negative polarity PD pulses versus applied voltage for both magnetic field-free and magnetic field-present measurements in point-plane configuration with distance variations from 20 to 60 mm.

Prior to the measurements, the test sequence was executed to make sure that the detected signal variation came from the applied magnetic field. A diagram of the switching sequence is shown in Fig. 13. The first part (I) of the PD-intensity recording refers to the magnetic field-free measurement and is followed by a test turn-off switching for around 10 s. After the high-voltage recovery (II), the PD intensity returned to the original level; then, the second switching operation refers to the turning on of the magnetic field, which resulted in a clearly elevated PD threshold (III) that is expressed by ΔN^−^. The last transition was to turn the magnetic field off to the stage (IV), where the PD level corresponds to the initial value. The influence of the magnetic field on the PD dynamics is shown in Fig. 14 on the comparison chart. The set of curves refers to the distance *a* of the HV tip from the ground; within each pair, there are traces that reflect the B = off and B = on stages. Number of discharges *N*^*-*^ corresponds to acquisition within 60 s. The plot reveals the linear relationship of the number of corona discharges with the rising voltages (from inception to certain stage prior saturation). The most important observation is that, in all cases, the PD intensity was amplified with the presence of a magnetic field. At corona inception level *U*_*0*_ (*U*_*0*_−PD inception voltage), this increase was smaller (reaching the broader span between the lines at *1.2U*_*0*_). It should be noticed that the observed PD inception voltage with the presence of magnetic field was lower by about 8% comparing to B = off stage. The lilac (9 kV) and red (16 kV) dots indicate the correspondence to the PRPD patterns in Fig. 12.

The markers of the time sequence profiles that are shown in Fig. 14 are indicated by the arrows. The plots clearly indicate the influence of the magnetic field on the corona discharge dynamics. Even at the inception level, the elevation of the discharge intensity can be detected.

**Figure 15:**
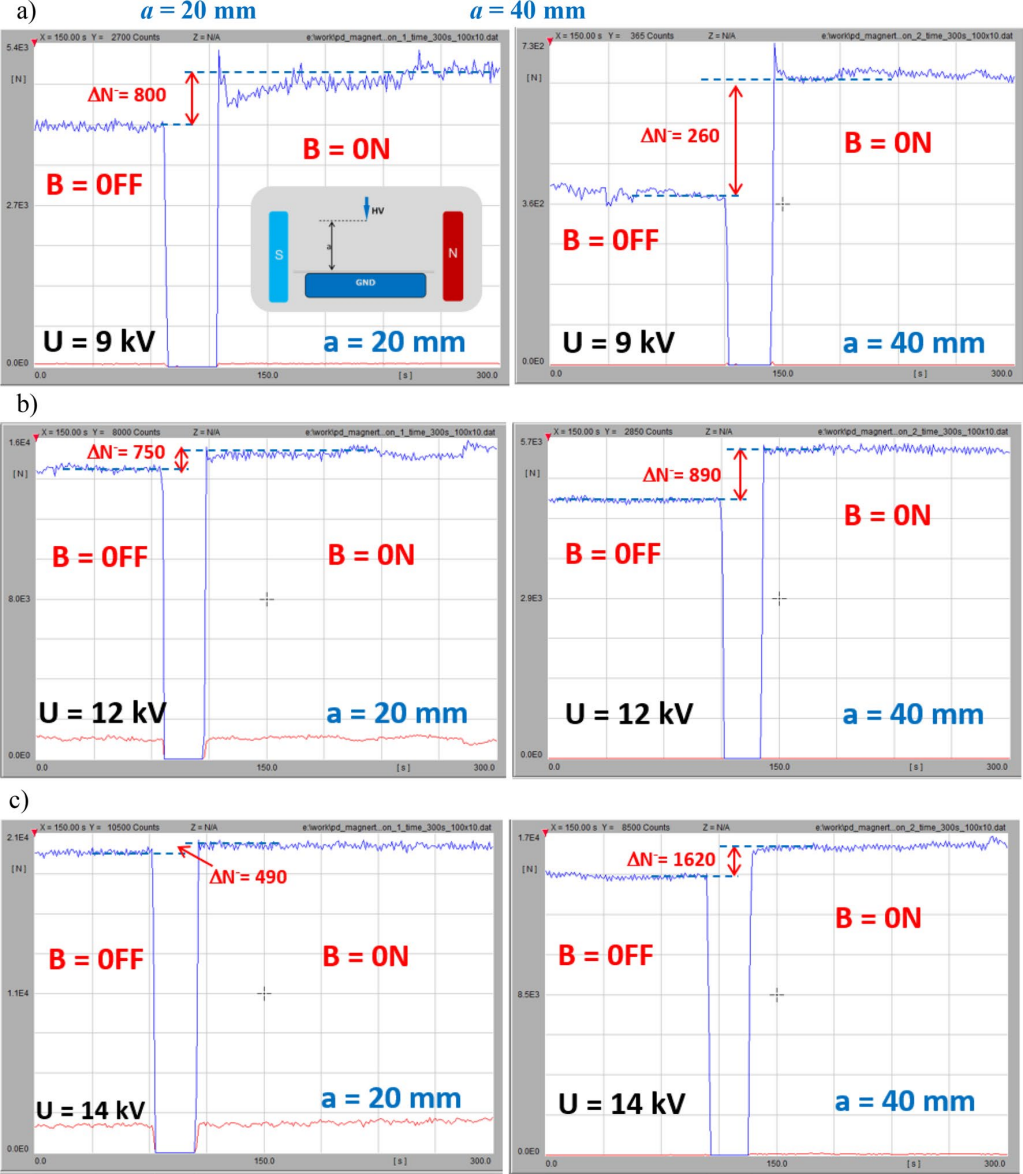
Time sequence B = off/B = on (*B*—magnetic induction) of influence of magnetic field on PD intensity (*N*—number of discharges) in corona point-plain arrangement for tip-to-ground distances of *a* = 20 mm (left column) and *a* = 40 mm (right column) at following voltages: (**a**) 9 kV; (**b**) 12 kV; (**c**) 14 kV.

The time sequence of the PD-intensity profiles at three voltage levels (9, 12, and 14 kV) as well as two interelectrode distances (20 and 40 mm) are illustrated in Fig. 15. Further increases in the voltage resulted in different dynamics (i.e., in the case of a distance of a = 20 mm, the PD number in the magnetic field-present state was elevated while increasing the voltage; however, the span expressed by ΔN^−^ was attenuated. Unlike for *a* = 40 mm, the voltage rise from 9 to 14 kV was associated with the strong dynamic growth of ΔN^−^ – from 260 to 1620, respectively). The above examples indicate that introducing a magnetic field leads to an amplification of the discharge intensity and an increase in the number of streamer channels. In the case of the corona, the enhancements refer to both the ionization zone and drift region. This effect can be confirmed by simulations presented in section “Conclusion”. Comparison of trajectories in crossed electric and magnetic fields for interelectrode gap distance *a* = 20 mm and *a* = 40 mm indicates elongation of the electron path, thus probability of discharge inception. Simultaneously, the residence and propagation time *t*_*p*_ in those two cases at B = 40 mT is extended from 0.5 to 1.1 ns.

The focus of the paper was to report the magnetic field modulated dynamics of partial discharges. Understanding the physical mechanism of the influence of the magnetic field on PD is a complex topic requiring further research. At this stage, some hypotheses can be put forward, which may explain the influence of the number of PD pulses, affecting also the PD magnitude. The higher number of PD pulses in the presence of magnetic field may be attributed to the lower inception voltage, which was observed in both cases, i.e. in the experiments with voids and corona. In addition to an electric field, the magnetic field component gives rise to an additional Lorentz force on charged particles. This force acting on free electrons in a crossed E × B field results in stronger acceleration compared to a drift only in an electric field, which will impact the energy of free electrons and enhance the ionizations of gas molecules. In this way, the magnetic field acts on the focused ionization zone, lowering the PD inception voltage and resulting in a higher number of discharges compared to B = off stage. The stronger acceleration impacts the number of collisions between the free electrons and the gas molecules, as well as the mean energy of the free electrons. Another hypothesis with respect to PD dynamics may be related to the fact that, due to different polarities, electron and positive ions will be deflected in opposite directions, extending the free electron path between collisions, thus causing more high energy electrons, leading to an increased ionization rate. In this scenario, deflection of positive ions will result in promoted free electron path, decreasing local recombination rate and thus increasing the probability of collision event. This effect will result in a higher number of PDs as well. The deflected trajectory of the discharge path under crossed magnetic and electric fields, visualized by imaging in^1^, results in an increase in the number of discharges, as measured in the presented experiment, due to the higher probability of discharge inception on a longer path. The perpendicular magnetic component is enhancing space charge concentration in the whole inter-electrode region, enlarging the possible ionization volume (since the straight drift path is modified to be more deflected and spiral-like), thus increasing the collision probability with ions and gas molecules. It may also result in an increase in the number of streamer channels, hence a higher PD number. The longer trajectory also means a longer residence time by charges in the interelectrode space, especially electrons that cause successive ionization events. This effect will impact also magnitude of the discharge. In a more macroscopic view, a propagating streamer channel consisting of electrons and ions will be impacted by a superimposed magnetic field. In future research, should be analyzed in addition to effects related to charge transport in the air, interfacial effects associated with the interactions on the PE surface, including surface emission and charge accumulation.

## Conclusion

This article presents original measurement methodology and detection approach to determine the influence of the magnetic field on the PD dynamics. The measurement were executed in the original setup which allowed for sensitive detection in the presence of weak magnetic field, unlike conventional PD measurement are carried out only in an electric field. The applied measurement technique allowed to detect the effect of magnetic fields on PDs in void and in corona mode. It was demonstrated that the interaction of electric and magnetic fields influences the dynamics of partial discharges in both configurations. In the acquisition approach time switching technique of magnetic field presence was synchronized with the PD detection. Combination of phase resolved images and time-sequence intensity diagrams allowed to visualize and determine quantitatively the impact of magnetic field on PDs.

It was shown in simulations, that the crossed electric and magnetic fields influenced the charged particle path, i.e. the elongations and turbulences of the electron beam trajectory. Due to the deflection that was caused by the effective Lorentz force, a longer pathway also led to prolonged residence time benchmarking while only being exposed to an electric field. At certain ratios of magnetic and electric fields, the presence of a magnetic field may lead to a focused and localized discharge spot, not a dispersed one as can be found in a field-free case. In this way, a magnetic field elongates the charge trajectories (i.e., for both electrons and ions) as well as enhances the energy; this is due to the acceleration in the combined fields. This can increase the probability of ionization, thus effectively increasing the discharge intensity; this was also confirmed by the measurements on both voids in the dielectric and corona. On the other hand, the twisting of a charge pathway may attenuate or even stop PD action. Such effects were observed for very tiny voids. It was shown that the impact of a magnetic field also depends on void thickness in the case of dielectric embedded inclusion (or interelectrode distance, as in the corona case). In the case of the gaseous inclusion in the dielectric material, there seems to be a certain critical inclusion geometry above which this effect is substantial. For example, the effect was not detected, and the attenuation of the PD number was not even noticed in the investigated void geometry and at magnetic field induction of 80 mT below the thickness of ca. 100 μm. The effect of a magnetic field on PDs was more predominant for the thicker void. Also, the number of discharges quantitatively rose more dynamically while increasing the voltage in the presence of both fields as compared to the reference case with only an electric field. This effect is attributed to the elongation of the charged particle trajectories and the enhancement of the electron energy due to acceleration. The presented study may contribute to measurement methodology, detection technique and the understanding of physical phenomena. It can raise the awareness of PD-intensity modulation that is caused by the presence of magnetic fields in most electric power devices. Thus, the effect of a magnetic field can be perceived as an additional modulation factor that influences PD dynamics.

## Data Availability

The datasets used and/or analyzed during the current study available from the corresponding author on reasonable request.
